# Visible beyond Violet: How Butterflies Manage Ultraviolet

**DOI:** 10.3390/insects13030242

**Published:** 2022-02-28

**Authors:** David Stella, Karel Kleisner

**Affiliations:** 1 Global Change Research Institute, The Czech Academy of Sciences, Bělidla 986/4a, 603 00 Brno, Czech Republic; 2 Department of Philosophy and History of Science, Faculty of Science, Charles University, 128 44 Prague, Czech Republic; karel.kleisner@natur.cuni.cz

**Keywords:** mating, communication, UV, reproduction, lepidoptera

## Abstract

**Simple Summary:**

Many animals, including insects, evolved sensitivity to ultraviolet light (UV). The presence of UV photoreceptors in the visual systems of many animals shows that UV-reflective traits are as important as other visible cues. Ultraviolet patterns on the surfaces of butterflies are among the most intricate UV-reflecting structures in the animal kingdom and they have been recognised and studied for many years. These patterns are often involved in intraspecific and interspecific interactions as signals of species identity or a cue reflecting the individual’s biological quality. In this review, we summarise the current knowledge about the significance of UV patterns in lepidopteran reproduction, including their role in mate choice and intrasexual competition. We focus on the mechanisms that produce UV colouration, discuss UV pattern variation in response to changing ecological factors, their adaptive function, and generally evaluate the evolutionary significance of communication in the ultraviolet spectrum.

**Abstract:**

Ultraviolet (UV) means ‘beyond violet’ (from Latin ‘ultra’, meaning ‘beyond’), whereby violet is the colour with the highest frequencies in the ‘visible’ light spectrum. By ‘visible’ we mean human vision, but, in comparison to many other organisms, human visual perception is rather limited in terms of the wavelengths it can perceive. Still, this is why communication in the UV spectrum is often called hidden, although it most likely plays an important role in communicating various kinds of information among a wide variety of organisms. Since Silberglied’s revolutionary Communication in the Ultraviolet, comprehensive studies on UV signals in a wide list of genera are lacking. This review investigates the significance of UV reflectance (and UV absorption)—a feature often neglected in intra- and interspecific communication studies—mainly in Lepidoptera. Although the text focuses on various butterfly families, links and connections to other animal groups, such as birds, are also discussed in the context of ecology and the evolution of species. The basic mechanisms of UV colouration and factors shaping the characteristics of UV patterns are also discussed in a broad context of lepidopteran communication.

## 1. Historical Sketch

Biologists are physically handicapped. In comparison with the organisms they study, their senses are often rather limited. This is most certainly the case with vision, whereby the study of animal vision began quite early and revealed a number of unexpected phenomena. Perhaps the most interesting and least explored has to do with the part of the electromagnetic spectrum that is normally invisible to humans, but most other organisms are visually sensitive to the ultraviolet (UV) part of the light spectrum. The wings of butterflies feature a wide variety of UV-reflective patterns, including the most intensive reflectance found in living organisms. In many species, these patterns show none or just negligible congruence with the pattern visible to the human eye [1]. These patterns can be produced by pigments, by structural means, or both. They are often species-specific and sexually dimorphic. Obviously, butterflies can use these patterns for intra- and interspecific communication.

The first comprehensive analysis of butterfly UV patterns was undertaken by Lutz [2], who published images of many species taken through a UV filter. His main goal was to indicate how a butterfly’s pattern may appear to another insect as opposed to the human eye. Unfortunately, Lutz in his study did not take into account the fact that insects are also sensitive to the visible spectrum, although some species display a weakness in the orange and red colour ranges. A subsequent study by Crane [3] analysed all possible methods of studying the UV spectrum and using UV photography; it surveyed in detail the UV patterns of 41 lepidopteran species. Crane also systematically described the colours (visible to the human eye) that correspond to the UV patterns and stated that the nature of all of these colours is probably structural. One of his conclusions, namely that UV patterns are rare among lepidopteran species, was probably due to his focus on tropical species.

Since then, our knowledge of UV patterns on the wings of butterflies and moths had expanded to include at least ten families of Lepidoptera [4,5,6], such as Pieridae [7,8,9,10], Nymphalidae [11,12], Riodinidae [13], Lycaenidae [14,15], Lymantriidae [6], and Papilionidae [3,16,17].

The aim of this text is to elucidate and review the functions and relative importance of UV patterns in a wide range of mainly lepidopteran species. The main idea was inspired by the ground-breaking work of Silberglied (1979), who was the first to attempt a comprehensive approach to UV in biology and likewise focused on butterflies.

## 2. Mechanisms of UV Pattern Colouration

A good understanding of the mechanisms of colour production along with their potential nutritional, physiological, and developmental costs is important for a holistic appraisal of the functions and evolution of these signals. Given that colour is the product of interactions between light, morphological structures, and eventually chemical pigments, selection that acts upon colour characteristics necessarily leads to changes in those underlying structures and chemicals. This is why we provide an overview of the mechanisms of UV production in butterflies.

The formation of UV patterns is based on small structures in the scales on butterflies’ wings. Although these structures are on a microscopic scale, they could be closely related to large-scale phenomena, such as the distribution of populations with different levels of UV reflectance. For this reason, it is crucial to describe the main factors responsible for UV colouration in animals. In general, there are two main colouration mechanisms, pigmentary and structural, which often simultaneously contribute to the wing colouration [18]. Pigmentary (or chemical) colouration is due to pigments that selectively absorb light in a specific wavelength range. Structural, or physical, colouration is due to the interference of light scattered on the nanostructures in the butterfly wing [19]. Interplay between the two colour mechanisms can influence the spectral composition of a signal and the information it conveys.

Wing surfaces consist of scales and each scale has a unique colour. A typical scale is about 200 µm long and 100 µm wide, but sizes in different species vary significantly. In most—but not all—cases, the scales form two layers, one consisting of larger cover scales, the other having smaller ground scales [20]. If we think of each scale as if it were a pixel in a digitised image, a pixel would contribute one point of colour to the overall image. In most cases, a scale consists of two laminae: a lower lamina, which is usually flat and solid, and an upper lamina, which is usually more complex and elaborate [21,22]. The upper lamina is structured by parallel longitudinal folds, usually called ‘ridges’, which are connected by orthogonal struts, so-called ‘crossribs’ [20]. The ridges are composed of lamellae and feature diffraction gratings known as ‘microribs’ [18]. The lower and upper laminae are joined by pillar-like trabeculae [23]. The ridges and crossribs are often organised irregularly, so that in the absence of pigments the scattering of incident light is wavelength-independent and results in a white colour on the scale [24]. 

Thin film layers are another relatively common variable feature in the structure of butterfly wings. Depending on the wavelength, these film layers can be constructive (e.g., UV iridescence in *Colias eurytheme*) or destructive [25]. Photonic crystals are specific single 3D or 2D network structures that reflect a specific light band depending on the angle of illumination and configuration of the crystal itself [20,26]. These structures, also known as ‘photonic structures’ [18], are species-specific and can be modified in various ways, which results in different optical and structural characteristics and different contributions to the wing appearance [25]. Moreover, if these structures are to produce maximally bright and/or achromatic UV signals, they need to be built with great precision and consistency, which is undeniably costly and hard for an individual to achieve [27]. Photonic structures are highly versatile, which allows for extravagantly varying shapes. In several cases, evolution led to the production of highly aesthetic, regular landscapes that produce spectacular iridescence [21,28].

Pigments do not produce colours per se. They function as filters. These pigment-based colours tend to be non-directional and diffuse, meaning they change very little with the angle of luminance or the viewing angle. The pigments responsible for the colouring of butterfly wings include pterins (Pierids), melanins (Nymphalids), and flavonoids (Lycaenidae). Most studies on UV colouration focus on pterins [29,30,31,32]. The main kinds of pterins are leucopterin, xanthopterin, and erythropterin [19,30]. Pterin pigments are deposited in small pigment granules, so-called ‘beads’, which enhance reflectance in the range of long wavelengths by increased scattering. In this way, they create a brighter wing colour and absorb diffuse reflectance in wavelengths below app. 550nm, which is revealed when the pigments are removed [28,33]. Leucopterin and xanthopterin only absorb light in the UV range. Moreover, these compounds amplify the iridescence properties of UV signals during wing movement [30,32]. The contrast in intensities is thus augmented in wing areas that reflect UV from the overlying lamellar thin films. These pigments also contribute to the reflection of what humans perceive as reds, oranges, and whites. Pterins are reported to be the most nitrogen-rich pigments known. In species whose growth and development are routinely limited, pterin deposition in the scale can thus be constrained, which is why colouration could be indicative of the quality of the individual [25]. An alternative view is that pteridines are the by-products of routine metabolic processes and their acquisition and expression is thus unlikely to be particularly costly [34,35]. 

When a scale structure follows a regular periodical arrangement, it gives rise to structural colouration. In pierids, structural colouration is found in so-called cover scales, where the ridge lamellae evolved into multi-layered structures that produce a highly directional iridescent colouration [21]. Some time ago, a detailed description of the four types of scales associated with physically produced UV reflectance had been proposed [5]. The structural colours optically vary and can range from diffuse, broadband reflections produced by incoherent scattering, interference, or diffraction, all the way to a narrow reflection of high purity and intensity [28,32] 

Illumination of the upper wings with white light can create blue light and iridescence, where the apparent colour depends on the illumination angle. This phenomenon is often discussed in the context of UV colouration and is known for its instance in *Morpho* species [36]. This optical effect is based on the taller ridges forming a multilayer interference mirror, sometimes called a ‘multilayer reflector’. These structures are overlain by clear scales, which, via diffraction, broaden the angle over which the iridescence is visible. It has been proposed that this effect enhances the perception of a signal by its intended receivers [37]. Iridescence peaking in UV wavelength occurs also in Pierids, mostly in the Coliadinae (e.g., *Anteos clorinde*, *Phoebis* sp., *Eurema candida*, *E. hecabe*, *Colias eurytheme*, and *E. lisa*; [1,21,38]), where it is produced mainly by ridges in scales, and its level is intensified via pterins, which absorb short wavelengths [19]. The distribution of iridescence in pierid wings varies among the various species. In most cases, iridescence is found only in males, but, in some species, the females are also iridescent. Detailed spatiotemporal relationships of iridescence were studied in *Colias eurytheme* [38], a butterfly which uses iridescence in its behavioural patterns mainly to communicate with conspecifics. Analysis of this behaviour also permits for some predictions regarding how signal senders and receivers need to be mutually oriented for maximal transmission and reception of this iridescent UV signal [39]. Angle-dependent structural colouration provides an opportunity to assess the spatiotemporal mechanisms of the dynamic relations between colour, behaviour, and environment because of the visibility of these structurally coloured ornaments, which depend on both the illumination angle—an environmental feature—and the observation angle that is on the receiver’s position [40,41]. For example, in *Gonepteryx* species the ridge lamellae are tilted with respect to the scale plane [21], whereby such tilting usually affects the visibility of the ornament in nature. In other words, the fast angular changes produce a strong flashing effect, which suggests that the UV ‘reflector’ creates a signal that strongly contrasts with the common colours and features of the environment [42]. The importance of inclusion of behaviour, wing pattern, and environment was highlighted by a study on avian plumage [43] and the main message of that research could be generalised. Overall, however, these kinds of optical interactions between scales and their potential consequences for colour signal production, perception, and evolution remain understudied. 

Several studies suggested that structural colouration serves to signal the quality of an individual. The key point of honest information regarding male mate quality is that only ‘cheating-proof’ signals, that is, signals which are costly or difficult to produce and/or to maintain, can be expected to be evolutionarily stable [44]. This also implies that the expression of these signals might be condition-dependent, i.e., only individuals with a good phenotype and good genetic equipment can develop and maintain an extreme version of this trait [27]. As a secondary sexual trait, UV colouration is more sensitive to developmental stresses than it is as a nonsexual trait. Structural colouration develops as a three-dimensional array. In most lepidopteran species, the development of colouration is restricted to a particular and restricted period of individual development, namely the metamorphosis, when it evolves from limited resources gathered during the larval stages. Structural UV can therefore function as a handicap [45] and indicate mate quality both in terms of adult nutrient status (phenotypic quality) and in terms of genetic quality, because it is dependent on the ability to acquire resources during the larval stage [27]. This is important because females receive fitness-enhancing nutrients during mating [46]. Interestingly, to the best of our knowledge only a handful of studies so far proposed mathematical genetic models of the formation and diversification of general colour patterns in butterfly wings. A majority of genetic and molecular studies focus on a local pattern, such as eyespots. For example, Yang et al. [47] in their study found an abnormal specimen of *Aglais urticae* that lacked the UV-reflective parafocal eyespots and exhibited changes of other pattern elements, which may be taken to indicate that there is a connection between the proximal and distal wing colouration and the pattern in its entirety is not a single character produced along the posterior/anterior axis. On the other hand, there are only several genetical studies which investigated patterns—including UV patterns—globally, although it is generally accepted that to understand the evolution and diversity of UV (as well as non-UV) patterns, one must investigate both the genetic and the molecular (developmental) traits with this in view. Such studies, however, are lacking [48]. 

In general, the mechanisms of UV and non-UV pattern formation are highly varied among species, and the variety of wing colours in butterflies is varied similarly. A unique colour signal of a species consists of a combination of structural and pigmentary elements. Angle-dependent reflectance measurements show that directional iridescence differs even between closely related species, such as Coliadinae (*Gonepteryx aspasia*, *G. Cleopatra*, *G. rhamni*, *Colias eurytheme*), Colotini (*Colotis regina*, *Hebomoia glaucippe*), Nymphalini (*Aglais urticae*, *Aglais io*, *Vannesa atalanta*), Papilioninae (*Parides* spp.), and Theclini (*Chrysozephyrus* spp.) [14,26,41,49]. Species-dependent scale curvature determines the spatial properties of wing iridescence. Narrow beam illumination of flat scales results in a narrow far-field iridescence pattern, while curved scales produce wider patterns. Restricted spatial visibility of iridescence probably plays a role in intraspecific signalling, as discussed above. Moreover, the position and type of pigments in the wings, the properties of the thin film (lamina), as well as the scale anatomy itself are all elements that, in conjunction, produce a species-specific UV colouration. 

When we take all of this on board, it becomes clear that there is no simple or general mechanism of wing colouration, and every species must be analysed on its own. In other words, we are far from understanding the details of the optics of butterfly colouration and we know that almost every species, and certainly those in different families, employ a variety of optical colouring methods. In many cases, pigmentary and structural colouration are combined in non-trivial ways [30,50]. The mechanism of UV pattern creation is an important part of understanding other crucial subjects too, such as communication in UV wavelengths. 

## 3. The Adaptive Function of UV Reflectance and Its Perception in Butterflies

### 3.1. Thermoregulation

Light reflectance plays an important role in thermoregulation, but while this holds for the visible and infrared light, it is not the case for the UV range because UV light has no thermal effect. Dark-coloured animals soak up warmth quickly, which increases their level of fitness for potential mating or nectar searching. This can improve the survival chances of ectotherms, such as butterflies [51]. Pigments, such as melanins, play a key role in the complex processes that serve to maintain thermoregulation in ectotherms. It has been suggested that lightness is driven by the need to protect from pathogens, while melanin levels are driven by UV radiation [52]. This ecogeographical rule of thumb, known as Gloger’s rule [53], applies only to endotherms, while, for ectotherms, it has been suggested that colour lightness in butterflies can be influenced simultaneously by thermoregulation and protection against UV radiation [54,55,56]. This depends on several factors, such as the life history of a specific species (e.g., its basking strategy) or the features of its environment, such as elevation [54]. The expression of pigments such as melanin and pterins could be genetically and/or developmentally conditioned [54,57], which is why it is crucial to consider a link between UV reflectance and this phenomenon, although pigment expression may at first sight seem tangential to our subject. For more on thermoregulation and melanisation, see e.g., [55,58,59,60]. 

### 3.2. Butterfly Communication

A large part of recent research of UV signalling focuses on birds with well-described mating systems, social systems, and ecological niches [61]. For most of the 20,000 species of butterflies, such information is unfortunately lacking [25]. Nevertheless, detailed empirical work in several species forms a solid base for investigating the use of UV patterns during social and sexual interactions among butterflies. Males tend to respond to the visual stimuli produced by a female and initiate courtship by presenting visual and chemical signals to which the female responds. Given that many butterfly species are sexually dimorphic with respect to UV reflectance (i.e., males and females of a species have different patterns), one potential function of these patterns could be sexual recognition. Many butterfly species have colouration that is highly similar in the visible light spectrum but highly distinct in the UV range. Extreme differences in the UV patterns of males of many closely related species suggest that they might be used for species discrimination by females, or even by males. On top of that, the shape and position of UV structures tend to be distinct enough to enable a relatively easy differentiation between species or subspecies. The iridescent UV reflectance produced by interference, with its high intensity, spectral purity, and abrupt flashing with a wingbeat, also seems to function as a long-range signal for various kinds of communications [39]. UV absorption may likewise be a signal. What is important is the contrast with the surrounding environment against which the wings are displayed in various interspecific ecological links.

#### 3.2.1. Intraspecific Communication

**Figure 1:**
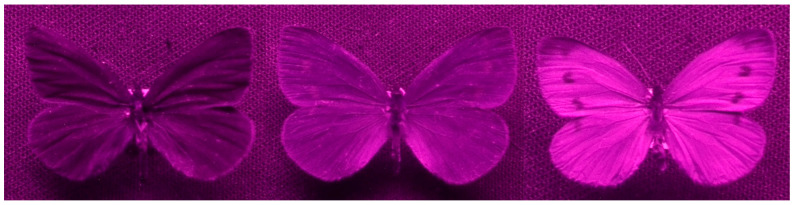
Various specimens of *Pieris napi* in UV light. The UV component of natural light decreases at higher latitudes due to a lower angle of the sun, which is why UV signals must be stronger to facilitate functional communication. Therefore, different specimens reflect different amounts of UV light.

Petersen et al. [62] were the first to demonstrate that the UV component of insect colour pattern might serve an intraspecific communicative function. They found that (UV-reflecting) zinc-white butterfly models were far more attractive to the males of *Pieris napi* and *Pieris bryoniae* than (UV-absorbing) lead-white models were. The females of *Pieris napi* [7,63], *Pieris bryoniae* [62], as well as other species of Pieridae, such as *Eurema candina* [38,64], *Pieris rapae* [33,65], *Pieris occidentalis* [66], and *Belenois zochalia* [67], reflect UV light more strongly than their conspecific males—these species thus display different degrees of UV sexual dichromatism. For example, Stella et al. [63] found a 25% higher level of UV reflectance in female *Pieris napi* than in their conspecific males (Figure 1), while Meyer-Rochow and Järvilehto [4] found a 35–40% difference of UV reflectance in this species. Nevertheless, this phenomenon, that is the females having higher UV reflectance levels than their conspecific males, in Lepidoptera is an exception [1]. It has been proposed that during the evolution of Pieridae there must have been a switch in the UV pattern of the sexes [1]. There are several hypotheses regarding the evolution of this sexual UV dichromatism. First of all, it has been suggested that females with higher levels of UV reflectance intensity are favoured by males, which is why the expression of this trait, i.e., high UV reflectance, in females could be adaptive and enhance fitness either in the context of male mate preference or in competitive interactions with other females [38]. A second explanation could be that the expression of the UV pattern is an incidental and non-functional result of genetic correlations between the sexes. This could lead to a penetration (integration) of genes that express the trait in females [68]. However, males of *Pieris napi* do not have highly UV-reflective patterns, due mainly to the presence of pterins, which decrease the level of UV reflectance and increase the degree of whiteness (i.e., the level of reflectance in the visible spectrum of light) in this species. Males are under a sexually selective pressure from the females for whiter wing patterns, i.e., patterns with a higher level of whiteness [69,70,71], which is probably why variation in UV reflectance levels in males appears relatively low in comparison to the females of this species [4,63]. It is expected that chemical removal of the pterin pigment on the wings of male *Pieris napi* would lead to an analogical appearance of UV reflectance as is seen in the females. 

The removal of pterin pigments has been tested on *Colias eurytheme* [32], one of the most studied butterfly species with respect to UV reflectance. Due to the different structures of the wing, the removal of pterin from the wing of *Colias eurytheme* led to a largely achromatic broadband white reflectance pattern and a decline in the iridescent properties of the UV signal. Therefore, it seems that in this case pterins increase the colour contrast as the UV flashes on and off during wing movement. Furthermore, the development of UV patterns in female butterflies could be explained by the hypothesis of *good genes*. During mating, males give females a costly nutrient investment in the form of spermatophore, which is used for somatic maintenance [46,72]. Thus, females select males according to their indicators (secondary sexual traits) such as colouring, the size of some structures [73], and their overall size [74]. This can potentially increase the survival or reproductive success of her offspring through, for example, a better ability to choose optimal microhabitats and nutritious plant foliage. Sexual traits are known to be highly variable [75]. In other words, individuals of a high genetic quality can be in good condition regardless of their developmental environment, whereas individuals of a low genetic quality prosper only in favourable environments [25,76]. According to the Hamilton–Zuk [77] hypothesis of parasite-mediated sexual selection, females should prefer males with the most elaborate sexual ornaments because the degree of ornament elaboration positively correlates with the males’ ability to resist various infections. By mating with elaborately ornamented males, females would thus acquire heritable parasite resistance for their offspring [77]. The level of UV reflectance could be one such dependent structure [1,78]. Moreover, different signals may correlate with different aspects of the bearer’s condition or quality [79,80], even with UV patterns that involve multiple colour mechanisms. The expression of these mechanisms may thus encode a number of distinct types of information about the signaller [81]. 

Some theoretical models show that, under certain circumstances, species could evolve multiple quality-revealing sexual ornaments [82], while other models propose that honest advertising should favour a single most revealing signal at the expense of other signals [83,84]. Despite these theoretical considerations, the hypothesis about the evolution of multiple quality-indicating sexual traits finds little empirical support in existing studies on Lepidoptera. Kemp [78] found that in *Eurema hecabe*, male UV brightness correlates with their body size and, interestingly, also with the size of their mate. This supports the hypothesis for the complexity of sexual interactions in polyandrous pierid butterflies, including, for instance, potential assortative mating [8,85]. Furthermore, one study found multiple quality-revealing sexual ornaments (nest features, plumage quality, ecoparasite load, body size) in bowerbirds, suggesting that bowers are an extension of the male phenotype that females can use to assess male quality [86].

In most butterfly species, males tend to take the initiative in mating behaviour [87]. Females may reject their advances for a number of reasons: the female may not be reproductively mature, had recently mated, or the courting male is not a conspecific and lacks the appropriate signal [1]. In some genera of Lepidoptera, such as the genus *Pieris*, the initial visual impression plays an important role in this behaviour. Previous studies showed that in *Pieris rapae*, UV reflectance is an essential and vital component for the activation of male courtship behaviour [87]. Female wing colouration (and not just UV reflectance) is undeniably a crucial feature in sexual discrimination [21]. This has been shown for instance in *Colias eurytheme*, where only the males display UV reflectance, which is therefore used by males for sex discrimination [1,32] and by females for mate assessment [88,89]. UV colouration was, in this case, suggested as the best predictor of whether a male will be accepted by a female for mating. Moreover, due to having a pterin-based structure, the wings of *Colias eurytheme* strongly flash during flight and it has been suggested that the frequency of these UV flashes falls within the female’s ability to discriminate the pulses of UV light [90]. 

Sex discrimination based on UV reflectance is possible and may be the case also in a number of other species, such as *Colias philodice* [8], *Eurema candida* [38], *Eurema hecabe* [38], *Pieris protodice* [4], *Pieris rapae* [64,91] *Eurema lisa* [21], *Hypolimnas bolina* [12], *Pieris occidentalis* [66], *Belenois zochalia* [67], *Chrysozephyrus* sp. [14], *Polyommatus icarus* [34], *Polyommatus andronicus* [92], *Bicyclus anynana* [93], and *Heliconius cydno* [94]. Given that the size of the wing area dedicated to UV-reflecting scales, the overall shape of the UV pattern, the level of UV reflectance, as well as the perceived hue and saturation usually differ considerably between the sexes, they can be employed in sex discrimination. Although sexual dichromatism is widespread among butterflies, it is not universal. UV signals function within a wider context and cannot be considered in isolation. In many species, it is likely that only the visible part of the light spectrum is used in sexual recognition. This is most likely the case with the wing patterns of *Colias philodice* [67], *Anteos clorinde* [38], and *Pieris occidentalis* [66], or the specific behavioural patterns such as those observed in *Pieris rapae* [64,95]. In some species, sexual recognition is aided by olfactory cues—for instance, in *Bicyclus anynana* [96]—and many species probably rely on a combination of cues depending on the timing and distance. Given that the UV signals quickly become less prominent with distance—though their reach depends on the mechanism of UV colouration (Burkhardt, 1989)—olfactory cues can also be used to signal at various distances and over time [96]. In many cases, we find a species-specific shift from dependence on visual cues to chemical ones in intraspecific communication.

The *Pieris rapae* butterfly occurs in two subspecies: *Pieris rapae crucivora* and *Pieris rapae rapae*. UV sexual dimorphism is, however, only found in *Pieris rapae crucivora*, while *Pieris rapae rapae* have no UV reflective patterns on the wings of either sex [91,95]. Less than 5% of sexual difference in UV reflectance was found in the *P. rapae* subspecies and the difference that was observed pertains to the morphological structure of the wings. Males of the *crucivora* subspecies have beads on their scales (sometimes called ‘pigment granules’) that contain pterin, a UV-absorbing pigment, while female *crucivora* lack this feature [97,98]. In the *rapae* subspecies, on the other hand, both sexes have beads on their scales and thus do not reflect UV light [97]. Recognition of conspecific males of the *rapae* subspecies is based on a ‘flutter response’, a specific behavioural feature which deters approaching males from attempting to copulate with males. In other words, it functions as a ‘mechanical isolation mechanism’ against maladaptive copulatory attempts between males [98]. The *crucivora* subspecies uses the flutter response as well, but only as a redundant behavioural feature [97]. If intersubspecific mating occurs in the subspecies *crucivora* and *rapae,* hybrids are viable with a highly variable UV pattern in the females [99]. The flutter response was also described in *Pieris napi* [4,62], *Hypolimnas bolina* [100], and in *Colias eurytheme*, where it functions as a signal of unreceptive behaviour [1]. It seems that the presence of UV reflectance in certain specific species could be explained by Amotz Zahavi’s ‘handicap principle’ [45], which proposes that such signals are evolutionarily stable because their expression is costly. This is why they relate ‘honest’ information about their bearer’s ability to carry such costs. Due to their high nutritional demands, the cost of sexual signals—in our case, UV patterns—is disproportionately higher for low quality individuals than it is for high quality individuals [58]. In other words, males with a high level of UV-reflective patterns can be viewed as handicapped by shouldering these costs, but they evidently can cope with it and do survive [8]. It should be noted though, that explanations based on the Zahavi’s ‘handicap principle’ have lately been criticised for being based on erroneous theoretical assumptions, namely that signals are reliable due to their wastefulness (costs) and evolved under the special type of selection that favours waste rather than efficiency [101]. In light of the above, it may be preferable to treat the issue of honest UV signalling within the evolutionary framework of adaptive signalling trade-offs with no reference to the handicap principle.

In a series of experiments with *Colias eurytheme*, it was established that UV patterns play a critical role in female mating behaviour [1,8]. In particular, males of this species whose UV pattern was destroyed suffered a significant decrease in the number of successful conspecific matings [32]. Female preference for males with brighter UV patterns was also found in *Hypolimnas bolina* [76]. A key prediction of the honest signalling theory is that the expression of directionally selected sexual traits should tightly co-vary with the condition of the phenotype [25,85]. This phenomenon is known as ‘condition-dependence’ [102]. The lamellar arrays that produce UV patterns may thus be costly to produce and may be *sensu stricto* condition-dependent (Morehouse-[103]), which would contribute to the honesty of this signal [32].

Intrasexual competition between males could provide an alternative explanation of the function of UV reflectance in butterfly communication. Behaviourally, most female butterflies tend to be solitary, whereas males interact vigorously either when defending their territory or when fighting over potential mates. Long-distance signalling is thus necessary for males but not for females [104]. The ability to recognise other males and the clear signalling of one’s own sex by UV reflectance is thus advantageous for males in the context of agonistic and territorial behaviour as well as mate location [1,105].

In tropical species, the situation is different. Curiously, despite the striking colours of tropical butterflies, studies on UV reflectance in tropical species are few and far between. A handful of studies suggest that tropical butterflies have either partial or spot UV reflectance—that is, much less UV patterning than non-tropical species do. Crane’s study, which was the first to investigate large numbers of tropical species, concluded that UV reflectance in tropical butterflies is rare and the low percentage of UV-reflecting patches cannot have a significant effect on the insect’s eye. In short, in tropical butterflies, UV reflectance is unlikely to have adaptive value for communication [3,18]. Huertas et al. (unpublished data), however, do report such adaptive patterns in the genus *Eryphanis*, where the shape of small spots differs from the shape of their UV reflectance and could be used to discriminate between the species or subspecies in this taxon. Another example of an adaptive function of UV reflectance in tropical butterflies is the neotropical *Heliconius*, famous for their wing pattern mimicry [106,107]. Their yellow aposematic spots are highly UV-reflective and often viewed as a hidden channel of communication where species-specific signals are not detected by predators [108]. This led to a suggestion that the visual system and wing colouration co-evolved so as to facilitate communication, especially among conspecifics [109]. Only two studies, however, tested concrete intraspecific communication preferences in the context of the variation of UV reflectance in tropical butterfly species, and the use of UV traits in signalling between different species has not been specifically addressed as of yet. Regarding the former, Robertson et al. [110] discovered that female African tropical *Bicyclus anynana* choose their mates based on the size and UV reflectivity of the dorsal eyespot’s central white pupil. Contrary to this, a recent study shows that choosy *Bicyclus anynana* males notice these white UV-reflective pattern elements and mate with females that have them more readily than with females in whom these patterns are blocked [93]. Secondly, *Heliconius* males also use UV signals to choose their mates, which indicates a trade-off between natural and sexual selection regarding visual signals, between the reduction in the likelihood of confusion in courtship, and a maintenance of the advantages of aposematic colouration [111]. Moreover, it seems that the dorsal colour of some *Heliconius* species may have evolved through selection for aposematism as a type of protection from predation, while its ventral surfaces were selected for sexual signalling. 

In other tropical butterfly species, there is clear evidence of signal partitioning between dorsal and ventral wings [112], and that includes UV signalling. In *Bicyclus anynana*, dorsal wing characters are involved in sexual signalling while eyespots on the ventral side of the wing play a role in predator avoidance [110]. The same trend in the function of the two wing surfaces appears in pipevine swallowtail (*Battus philenor*), where the colour pattern on the ventral hindwings in both sexes acts as an aposematic signal that advertises distastefulness to potential predators [112]. Morpho butterflies have a flickering flash iridescence colouration ranging from blue–green to UV on the dorsal side too, which is involved in male flight patrolling [3,113], while cryptic colours on the ventral side seem to serve as protection against visual predators [114]. 

The quality of UV reflectance declines with age due to wing wear, scale loss, or damage. This can be used in mate assessment by females [89], as is the case for instance in *Colias eurytheme*, where structural UV colouration is a valid indicator of age [11]. The nymphalid butterfly *Anartia fatima* is unusual in that both its UV reflectance and visible colour change with age: in both sexes, the yellow UV-absorbing bands become white and UV-reflecting. Older, UV-reflecting females are then more attractive to mate-seeking males [67].

Studies on the evolution of UV patterns in Lepidoptera are few and far between and only a handful of articles so far offered a discussion of UV reflectance from an evolutionary perspective. Research on the molecular phylogeny (mitochondrial DNA) of Lepidoptera indicates that, within the genus *Colias*, the oldest species in Europe are the Scandinavian ones and it is from the north-west of Europe that *Colias* butterflies spread south and east, eventually forming nine species. This analysis seems to indicate that UV reflectance evolved several times (a polyphyletic trait) within the genus (and each clade), which is consistent with the hypothesis that UV traits are subjected to intra- and/or interspecific selection [115]. Brunton’s study, however, was based only on 12 species and, in light of more recent studies [116], its results cannot be considered fully reliable. UV patterns in genus *Gonepteryx* also lend themselves to a phylogenetic mapping of the evolution of this trait. Nekrutenko’s [117] study, which was based on the investigation of UV patterns, showed relationships between the *Gonepteryx* species, and its results were mostly congruent with later molecular studies [118,119]. Another recent study based on both mitochondrial and nuclear DNA describes the phylogeny of *Gonepteryx* butterflies in even more detail [120]. Moreover, this paper used UV patterns in its analyses as a covariate and showed analogous spatial trends in UV patterning as in the *Colias* genus. 

#### 3.2.2. Interspecific Communication

Related species are often sympatrically distributed, which is why UV patterns can be used in species recognition. This is of importance especially in relation to avoidance of interspecific mating and through character displacement, also in the context of sympatric speciation [121]. In many Pieridae species, only males exhibit UV reflectance and, because male courtship harassment carries costs for females [122], UV patterns that distinguish the females of related sympatric species could reduce interspecific male harassment and thereby increase time available for egg laying [38,67,105]. In *Eurema*, *Phoebis*, *Colias*, and other genera, there are marked interspecific differences in male UV-reflective patterns among congeneric sympatric species. The females of *C. eurytheme*, for example, accept conspecific males who have a strong UV reflectance. Behavioural observation showed that males with obliterated UV patterns mate less frequently than control males do [1,8]. In *C. eurytheme*, it seems that UV patterns play a role in species recognition. In contrast, the females of *C. philodice* (a species where males are UV-absorbing), do not discriminate against conspecific males who were experimentally adorned with *C. eurytheme* UV-reflecting wing patches. Thus, there is no reason to expect that all *Colias* species use UV patterns in communication and mate selection. It is quite clear that, in some species, UV signalling does not play a dominant role in communication. Moreover, Brunton and Majerus [105] analysed the intra- and interspecific variation of UV patterns in a number of *Colias* species. One might expect very little variance in UV reflectance between specimens of the same species. Nevertheless, a study comparing inter- with intraspecies differences in a number of *Colias* and *Gonepteryx* species concluded that intraspecies variation was so high in several European species that neither butterflies nor their predators were likely to be able to differentiate between species based on their UV patterns [105]. This is also why UV patterns are unlikely to play an important role in mechanisms of reproductive isolation in these butterfly species. 

Thus, UV patterns clearly cannot be considered in isolation and, with respect to communication, other channels must be taken into consideration. For instance, *C. philodice* relies entirely on olfactory cues [8,123]. In other words, while UV signals can function as an isolating mechanism for some species, such as *Pieris napi* [1,124] or the *Actinote* genus [125], where they reduce the risk of hybridisation, in other species, such as some *Colias* butterflies, UV reflectance probably plays a minor, if any, role in this type of interspecific communication. 

As described above, the wing patterns of some butterfly species involve hidden features such as UV patterns. For this reason, UV reflectance patterns are used as taxonomic tools to distinguish between species, thus dispensing with the need for complicated morphological and genetic analyses. Pioneering taxonomic work based on the UV reflectance of *Gonepteryx* species was done by Nekrutenko in a number of his studies [9,117,126,127,128,129]. He hypothesised that UV patches could be a useful taxonomical trait. To this purpose, he described a number of characteristic features in the terminology of ‘hidden’ UV wing patterns on the wings of *Gonepteryx* species and was most likely the first to describe gynandromorphy in the UV spectrum. Bozano et al. [119] recently proposed the first molecular-based phylogeny of the genus *Gonepteryx*, which changed the taxonomic status of some traditional subspecies to a species level. More importantly, this study and a number of other recent works treat *Goneptryx* UV patterns as a possible diagnostic trait of a taxonomic value [105,119,120,130]. Further studies used UV patterns as an auxiliary taxonomical method for *Papilio* [131], *Lycaena* [132], and several *Colias* species [116]. 

Already decades ago, Ferris believed that UV photography is the most useful and cheap method for complicated taxonomical analysis. He assigned a number of species/subspecies to various colour groups [133,134], which later genetic analysis indeed proved to be separate species or subspecies [135]. Furthermore, analysis of UV reflectance patterns on the wings revealed significant differences in two species of swallowtail butterfly (*Iphiclides feisthamelii* and *Iphiclides podalirius*). This study was supported by genetic (nuclear DNA) and morphological (male and female genitalia) analyses to underpin the importance of UV reflectance as a taxonomical tool [136]. Even more recent findings support the taxonomical significance of UV reflectance in south American *Eryphanis* butterflies (Huertas et al, unpublished data). Although UV patterning is, in taxonomic studies, employed relatively rarely, all of the abovementioned sources confirm the importance of this feature as a taxonomical tool that does not require complicated morphological analysis.

Butterfly UV patterns could also serve as a decoy or warning colouration (aposematism) for various species of birds, thus playing an important role in prey–predation interactions [6,67,137]. Brues [138] was one of the first researchers to hypothesise about UV patterns functioning as a Batesian or Mullerian mimicry. Without any field observation, he claimed that mimicry is the main force driving UV pattern formation in various butterfly species. Remington [125] found that African butterfly models and mimics resembled one another in UV-reflecting patterns more than New World models and mimics did. He suggested two explanations: African predators see in the UV spectrum better than new World predators do or, alternatively, mimicry complexes in Africa had more time to evolve. Selection against UV patterns may be expected because reflected UV light could attract avian predators [139]. The hypothesis of a substantial negative selective pressure against UV patterns finds support in the fact that nocturnal species have UV patterns more frequently than diurnal species [6], which move around more at times when visually hunting predators are active. In species where there is sexual dimorphism of UV patterning, females tend to be more cryptic or mimetic than males. This should decrease the risk of predation; in other words, it should function as a protective pattern. Males, meanwhile, tend to preserve the ancestral pattern of species [69]. That, however, was proven only for some species [105,115], while, for instance, *Pieris napi* exhibits an inverse phenomenon and its females are more visible in UV light than the males are. 

Cryptic patterning is crucial not only for butterfly images but also for lepidopteran larval stages. Church et al. [140] measured the reflectance spectra of lepidopteran larvae and their natural backgrounds. Their results indicate that many caterpillars match the leaf background in both UV and visible wavelengths and are thus cryptic over this entire wavelength range. Furthermore, resting adult butterflies fold their wings to join them in an upright position. In most species, the reverse side of the wings is not UV-reflective [21,124] and thus, predators cannot use UV patterns on the wings of resting butterflies for prey detection [6]. 

Studies of UV patterns include the most widely quoted example of evolution in action: the story of industrial melanism in *Biston betularia*. In light visible to humans, the speckled *typica* form appeared cryptic when seen against the background of foliose lichen, whereas the dark *carbonaria* form was conspicuous. Under UV light, the situation was reversed. Foliose lichens absorb UV light and appear as dark as the *carbonaria*. *Typica*, on the other hand, reflected UV and was conspicuous. Against crustose lichens, the *typica* was less visible than *carbonaria* in both visible and UV light [141]. As is well known, during the Industrial Revolution in Britain, the *typica* form in urban areas was largely replaced by the *carbonaria* form. As pollution later decreased, the *typica* form once again became dominant. The abovementioned study by Majerus et al. [141] somewhat complicates the story but also highlights the importance of framing the investigation of UV reflectance in prey and predators in an ecological context. 

A number of studies suggested that butterfly colouration, with high frequencies of oranges, yellows, and whites, is in fact aposematic, i.e., that it warns potential predators that the butterfly in question is protected [142,143]. This finds support in the fact that a number of butterfly families include species with a wide range of palatability, so their patterns may indeed have an aposematic function [137,144]. So far, however, the issue of aposematism and UV patterns has been addressed by only a handful of studies, whereby some research indicates that the aposematic colouration hypothesis may be wrong. For instance, a study on great tits (*Parus major*) showed that the birds were incapable of learning to avoid unpalatable prey items regardless of whether the prey reflected or absorbed UV light [145]. In other words, birds exhibited no strong avoidance of UV-reflecting prey. This is analogous to the finding that a removal of UV reflectance had no significant effect on the foraging behaviour in avian merkwelt [146]. Nevertheless, UV signals should not be considered in isolation and the entire spectrum of wing patterns needs to be examined [147].

In a slightly different context, however, highly reflective UV patterns could serve as effective defences against predation. Gopal Murali [148] demonstrated that butterfly movement accompanied by striking colouration, which dynamically changes over time, is a functional protection against predation. It probably works by causing predators to misinterpret the prey’s location. As well, iridescence, which leads to fast colour changes, could serve as a form of dynamic disruptive camouflage based on the same principle [149]. Although this was tested in the context of wavelengths visible to humans, UV patterns—which can function as a strong signal in the visual merkwelt of organisms sensitive to UV light—could be included in this functional explanation of UV pattern configuration in a parallel fashion. Robertson et al. [110] found highly reflective UV spots on the dorsal side of *Bicyclus anynana* wings, but, due to the central placement on the wing and their inaccurate mimicry of vertebrate eyes, it is unlikely that such patterns effectively deflect attacks to the wing margin. Contrary to this, Prudic et al. [150] found an association between increased butterfly survival and prominent eyespots in *Bicyclus anynana* in the context of predation by certain invertebrates, specifically praying mantids which cannot sense UV light. Consideration of the sensory capacities of predators is therefore crucial in such studies. Moreover, an experiment with UV eyespots in *Lopinga achine* had demonstrated that natural marginal eyespots on butterfly wings can deflect predator attack to these nonvital parts of butterfly bodies in low intensity light with prominent UV components, that is to say, in light conditions which emulate dawn or dusk—i.e., the times of the day when the butterfly’s avian predators are most active [151].

Some kind of trade-off between the development of signals aimed at conspecifics and signals aimed at predators undoubtadly also affects UV wing patterns. This conflict between natural and sexual selection is traditionally described as follows: if predation is a relatively stronger selective force than sexual selection, colouration will be more conspicuous for aposematic species and more cryptic for camouflaged species. If, on the other hand, predation poses a relatively smaller threat, colour patterns will be closer to the optimum for mate choice [40]. This was studied in *Heliconius* butterflies where males use UV signals for mate choice, indicating that the conflicting forces of natural and sexual selection do affect visual signals. Rather, they reduce the cost of confusion in courtship and maintain the advantages of a warning colouration [111]. Specifically, in *Heliconius,* it seems likely that their dorsal colours evolved via selection for aposematism as antipredator protection, while their ventral wing surfaces were selected for sexual signalling.

The theory of sensory drive claims that signalling systems should evolve so as to optimise the transmission between senders and intended receivers while, if at all possible, reducing visibility to eavesdroppers [40,76,152]. Highly directional and iridescent UV reflectance could be an example of such a trade-off. In other words, the expression of such visual sexual signals is generally thought to represent a balance between the effects of sexual and natural selection [76]. These signals require high conspicuousness—because to successfully compete for mates, the male must advertise certain qualities—but also relatively low visibility to predators. For example, Hastad [153] theorised that certain species attune their signalling to the visual systems of intended addressees, i.e., their conspecifics, while reducing the colour contrast to the background in the spectral ranges that their predators are most sensitive to. In other words, organisms use colour signals that are more readily seen by their conspecifics’ (and thus also their own) visual system than by their predators, thus creating a directed communication channel for displaying male quality. Moreover, Mullen and Pohland [154] described an association between the position of UV reflectance maxima and the possession of UV cones in almost one thousand bird species, which strongly indicates that the perception system is interspecifically tuned and, moreover, that the prey can hide its communication within its own UV reflectance maxima. The same phenomenon for covert communication appears, for example, in the mimic *Heliconius* butterflies [108,109] where UV signals are used in mate selection by females. A similar phenomenon has been hypothesised in *Hypolimnas bolina* [76], where the maximally bright UV reflectance of white spots surrounded by shimmering blue scales on the black upper side of the wings of courting males can only be seen from a highly restricted angle. These angularly restricted signals have a narrow angular reflectance: they are visible only from about 20° range of the above-wing viewing position. Specific behaviour also plays an essential role in the enhancement of signal transmission to females of this species. As a consequence, the male UV pattern is clearly visible to *H. bolina* females but not to avian predators, which is clearly advantageous. 

To sum up, UV patterns are subject to a dual challenge of maximisation of signal transmission to conspecifics and concurrent minimisation of possible detection by visually orienting predators. The theory of sensory drive predicts that such signals should be designed or transmitted to some degree privately [40]. Some authors describe this phenomenon using the term ‘private communication channel’ [155,156]. 

### 3.3. The Effect of Environment on UV Reflectance 

A number of spatial and temporal variables affect various functional traits in the life history of Lepidoptera, such as their body size, flight performance, wing shape, mating performance, or overall fitness [157]. These variables include temperature [158,159,160], altitude [58,161,162,163], latitude [164,165], and net primary production [166], but their effect on UV patterns is studied rarely if at all. 

**Figure 2:**
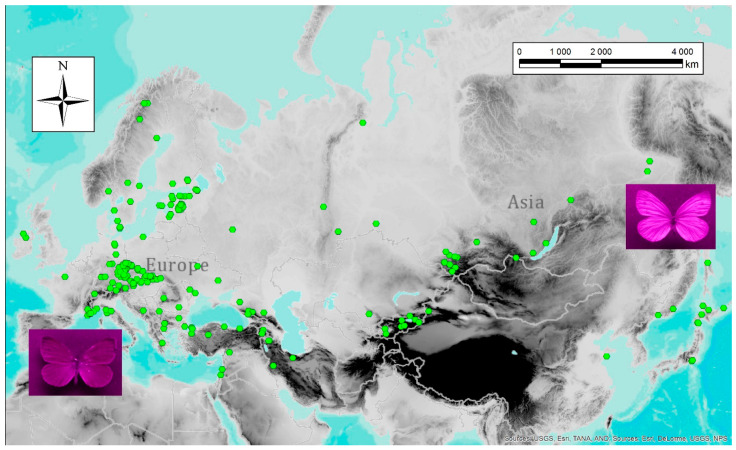
Locations of the 407 specimens of the green-veined white (*Pieris napi*) from the Palaearctic region from [61]. Female *Pieris napi* generally exhibit a less intense UV reflectance in western and southern regions of the Palaearctic.

The extent to which UV patterns are genetically fixed or flexible and changing in response to environmental pressures is not understood in detail but most insect species do display a degree of phenotypic plasticity, which enables them to adapt to particular environmental conditions. Nevertheless, some studies did investigate the purpose of UV patterns in the context of environmental variables. They worked, for instance, with latitude and variation across a broad latitudinal space. In fact, latitude has been used as a proxy in a series of macroecological studies: for instance, Meyer-Rochow and Järvilehto [4] tested the effect of latitude on dorsal UV wing pattern and revealed significant differences between the studied specimens. They, as well as a number of other researchers, found that females of *Pieris napi* from the northern part of the studied range had more prominent UV patterns, whereas the males tended to remain more phenotypically constant and exhibited a smaller degree of UV pattern variability [69,105,167]. A similar trend was found in bumblebees: those living at higher latitudes reflect UV light more strongly than those inhabiting low latitudes [168]. One of the most likely explanations of this phenomenon is that the UV component of natural light decreases at higher latitudes due to the lower angle of the sun, which is why UV signals must be, under these conditions, stronger to facilitate functional communication. This is congruent with the results of our study that analysed over 400 specimens of *Pieris napi* from the entire Palearctic region [63]. Generally speaking, localities characterised by extreme values of several variables, such as high seasonality of temperature and low overall temperatures, favour *P. napi* butterflies with stronger UV reflectance (Figure 2).

Large-scale environmental variables were explored in a study that focused on *Gonepteryx rhamni* butterflies [130]. This study found a systematic correlation between an increase in the relative area of UV colouration, increased temperature and precipitation, and decreasing latitude at which the specimens were collected. In this case, UV patches might serve as protection against UV light; such function has been proposed for flowers, where highly reflective pollen can function as UV protection after anthesis [169]. A follow-up on the abovementioned *Gonepteryx* study that worked with 320 specimens of seven species revealed that the shape of a UV patch (signalling trait) is more asymmetric than the shape of wing venation (non-signalling trait). Unfortunately, the correlation between the environment (latitude) and wing shape (fluctuating asymmetry) was not significant in all cases [170]. In other words, the study concluded that at least as far as *Gonepteryx* butterflies are concerned, UV patches are not a condition-dependent trait. 

A study that compared two subspecies of *Pieris rape* revealed a significant gradient in wing UV reflectance along the east–west axis [95]. UV reflectance of the wings was higher toward the east, culminating in a Japanese subspecies (*Pieris rapae crucivora*). This variation is probably due to differences in inter-subspecies mating between UV-reflecting East Asian butterflies and ancestral UV-absorbing European butterflies [171]. Furthermore, only a weak relationship between UV pattern and habitat properties was found in 54 species of *Colias* butterflies [116]. In that study, the different species tended to have different life histories, which made it difficult to associate UV reflectance with some general ecological variables. Recently, Dalrymple et al. [172] investigated multiple dimensions of macroecological variations and their relative importance for butterfly colouring. They found that variables related to energy and resources in the environment consistently function as the most powerful predictors of butterfly colouration. Their complex study, however, analysed the entire spectrum from 300 nm to 700 nm and paid no specific attention to UV patterns. Unfortunately, it dealt only with one relatively small region (Australia) and its results are hard to generalise to other areas.

It is generally accepted that UV patterns correlate with UV radiation in the environment. Such association was described, for example, in a study on *Eurema hecabe* [78], where incident light was manipulated in such a way that the mating rates of *E. hecabe* were six times higher in a UV cage (where UV radiation was present in the cage) than in a non-UV cage (where UV radiation was reduced). Moreover, because the male UV signal was dulled (but not completely eliminated), these results indicate that females prefer to mate with males that have more brightly coloured wings. All in all, this study confirms the importance of the configuration and level of UV irradiance in lepidopteran life history and its environment. Beckmann et al. [173] produced a UV radiation dataset at high resolution to emphasise the importance of this factor in many ecological processes and the geographical distribution of organisms. Their dataset is congruent with known patterns of global UV distribution, but it also revealed further details of spatial and temporal relationships that could be useful for ecological studies. Although a number of direct and indirect effects of UV radiation has been described, to the best of our knowledge UV radiation has not been systematically related to the configuration of organismal UV patterns (the sole exception being one recent study on UV patterns in marsh marigolds, the *Caltha palustris* [174]). Such links could help disentangle the complicated macroecological relationships between correlated variables, such as temperature and precipitation, and help reveal patterns in the distribution of functional response traits, such as UV patterns. Another interesting phenomenon related to ambient UV light is found in the *Anolis* lizards, where cosmopolitan species are more variable in colouration. Different morphs of *Anolis conspersus* live in habitats characterised by different light conditions and it seems that morphs with greater colour variation tend to live in habitats where light conditions are more varied [175]. UV light may the drive colouration of lepidopteran species or subspecies in the same manner, but studies unfortunately have not yet been undertaken. 

UV colouration is also significantly affected by another developmental factor, namely temperature. Research in *Drosophila* indicates that even relatively small departures from optimum developmental temperatures can result in temporary adult male sterility. In *Colias eurytheme*, the stressful effect of thermal manipulations was confirmed by a study that described a decreased rate of pupal development and a consequent impact on the expression of UV wing patterns [27]. Congruent with these results are also the conclusions of research on the seasonal forms in *Bicyclus anynana.* It showed that courtship, mate preference, and ornament UV reflectance in this species change depending on developmental temperature, which suggests that seasonal temperature variations lead to a complex polyphenism in mating behaviour and morphology [176].

Additionally, UV patterns (in fact, pterins) in various butterfly species—such as *Colias eurytheme*, *Pieris rapae*, or *Eurema hecabe*—may signal the individual’s condition via their correlation to larval adaptation to the host plant quality (mostly nitrogen availability) [177,178,179]. Adult *Colias eurytheme* males exposed to poor quality larval environments are not only smaller overall, but also exhibit duller and more angularly restricted UV reflectance [27]. In *Eurema hecabe*, low condition males even resemble high condition females [180]. Variability in the UV wing patterns of *Polyommatus icarus* can be due to differences in the plants available as larval food, even under otherwise identical rearing conditions among individuals from the same parents [92,181]. In this case, flavonoids are the main driver that shifts the configuration of UV patterns in *Polyomnatus* butterflies. Previous research had shown that, in *Pieris* butterflies, the degree of UV reflectance depends on the presence of pterins, the UV-absorptive pigments. For instance, in green-veined whites (*Pieris napi*), individuals capable of using the environment more effectively and/or those living in areas richer in nutrients reflect more UV light than those from warmer areas, which are, however, more suitable for breeding [63]. In all these cases, acquisition of larval resources seems to be the main determinant of the condition of individuals.

Bird feather patterns and structure are usually viewed as condition-dependent secondary sexual traits; in this respect, they are similar to butterfly wing patterns [182]. Like avian populations, lepidopteran populations exposed to different environmental conditions seem to face different selective pressures. These result in differences in wing structure and colouration, which are especially apparent in species with a large geographical distribution. In general, UV patterns are considered a condition-dependent feature and seem more sensitive to developmental stress than putatively non-sexual traits. The precise nature of links between the development, environmental conditions, and variations in UV patterns, however, remains unclear. 

## 4. Butterfly UV Vision

Research into the biology of UV photosensitivity and vision has, over the past decade, made much progress [183]. Researchers approach the subject from various angles including genetics, ecology, behaviour, and evolution. To understand the UV vision of studied species is of essential importance if we are to disentangle the complex biological mechanisms involved. It is also clear that, in this particular area, we cannot limit ourselves to understanding the phenomenon in question, i.e., UV-sensitive visual systems, merely from our human perspective. That would necessarily lead to erroneous conclusions.

Differences between butterfly eyes studied so far suggest that the capacity to discriminate colours varies among species and is driven by visual tasks determined by the species’ behaviour and habitat [184]. The compound eyes of butterflies consist of a number of anatomical units, ommatidia, which are organised to form a more or less regular hemisphere. Ommatidia are usually present in three types, which are characterised by their photoreceptors and pigments. They are rather complicated but, in brief, we can say that a facet lens and crystalline cone jointly project incident light into a photoreceptor. A butterfly ommatidium (retina) contains six or more photoreceptors [185]. Their light-sensitive organelles, rhabdomeres, jointly constitute a fused rhabdom, a long cylinder that acts as an optical waveguide. The special feature of many butterfly eyes is the presence of a thin reflective layer, tapetum, located near the rhabdom [186]. Visual perception thus starts with the absorption of a photon by visual pigments located in the rhabdomere. Incident light propagates along the rhabdom and, when it is not absorbed, it is reflected by the tapetum. Light then travels in a reverse direction and, if not absorbed, eventually leaves the eye and it can be observed as eyeshine—also known as ‘eye glow’. The eyeshine phenomenon allows for in vivo optical characterisation of individual ommatidia [187]. 

The visual pigments, rhodopsins, are opsin proteins combined with a chromophore. Isomerisation and subsequent photochemical processes form the chemical basis of photon absorption. The general organisation of butterfly colour vision is similar to that of honeybees and bumblebees, which are probably the most commonly studied subjects in insect vision studies [188,189]. The butterfly vision system is, in most cases, based on three photoreceptor classes, with maximal sensitivity in the ultraviolet (UV), blue (B), and green (G) wavelength range [190]. Nymphalids are, in this respect, an exception because they have a tetrachromatic colour vision system [191,192]

The dorsal rim is a special area of compound eyes: it consists of a few ommatidia whose photoreceptors have a very high polarisation sensitivity [184], which, in Lepidoptera, is restricted to UV polarisation [193]. For instance, the migratory monarch butterflies (*Danaus plexippus*) use this structure for flight orientation based on polarised UV light [194]. For a more detailed description of butterfly compound eyes, see [184,185,195].

The distribution of receptor types is, however, highly varied: it differs both among species and between the sexes. The reasons driving such diversity in butterfly colour vision are not fully understood but are probably linked to the typical life histories of individual species. In many species, colour receptors are not uniformly distributed and the patterns of expression of visual pigments across the eye can correlate with their life-history, or as it is sometimes called, their ‘visuoecological lifestyle’. Some experts in this context proposed the ‘efficient coding hypothesis’, which suggests that the retina evolved so as to efficiently encode a given visual environment. In other words, it evolved so as to minimise the redundancy of information carried by different neurons [196,197]. 

This convergent co-evolution was studied, for example, for flowers and their pollinators [198] or between three closely related sympatric species of the dung beetle and their environment [199]. The small white, *Pieris rapae crucivora*, is a prominent example of a species with a heterogenous retina. In this species, we find three types of retinas. The main difference between female and male photoreceptors is that the eyes of male *P. rapae crucivora* contain a fluorescent pigment that acts as a specialised UV filter and functions as a narrow-band violet receptor [187], which probably improves the discrimination of UV contrast. In the eyes of males of another *P. rapae* subspecies, the *P. rapae rapae*, this fluorescent pigment is, however, absent [28]. It was therefore hypothesised that the Japanese *P. rapae* subspecies *crucivora* evolved an improved discrimination of UV-visible patterns in the wings whose contrast is enhanced by scattered beads. A similar same-sex discrimination trait occurs in *Heliconius* butterflies [108,200] or in the *Asterocampa leilia* [201]. These differences in vision are due to gene duplications followed by mutations that affect amino acids in the opsin (visual protein) and lead to variations in spectral sensitivities between species [202]. For example, both *Pieris rapae* and *Lycaena rubidus* have gene duplications on the B opsin, but these duplications led to different extended visual spectrum ranges and to a sexual dimorphism in *Lycaena* eyes [202]. Another way in which vision can be altered is by application of spectral filtering in ommatidia [203].

The difference in the organisation and configuration of ommatidia is often pronounced in short-wavelength photoreceptors, i.e., in UV photoreceptors [204]. This led to suggestions that, for example, in the *Heliconius* complex or in the Pierids, the visual system and UV pattern co-evolved and might thus function as a privileged, ‘private’ channel of communication [184,203,205,206]. For example, it has been demonstrated that *Colias eurytheme* has a high ventral acuity and relatively larger eyes than other species. A possible adaptive explanation of this phenomenon may be that this facilitates hidden communication by UV signals produced by males [207] or females [208]. In general, species that use their UV patterns in intraspecific communication tend to exhibit some specific alteration of UV receptors [209], although it must be borne in mind that the importance of UV perception undoubtedly extends beyond mate recognition. 

Indeed, not all communication in butterflies is based on visual signals. Silberglied (1978), for example, explored the importance of visual contact of butterflies during mating of several species and noted that no chemical stimuli are needed to elicit mating behaviour. However, this is only the case in some species. For instance, for *Colias eurytheme*, olfactory cues are more important than visual signals: the initial mating approach requires visual stimuli, whereby even indistinct ones suffice, but further mating depends on chemosensory cues [105]. In other words, final recognition of females by males is made chemically. For example, *Pieris napi* emits citral, a sex pheromone that positively affects female willingness to mate [210]. 

## 5. Discussion and Conclusions

The remarkable colours of butterflies and their possible functions have been attracting special attention of biologists ever since the time of Darwin (1909), Wallace, and Lorenz (1962). All three of these respected scientists arrived at basically the same conclusion, namely, that in the varied manifold of various environments, striking colours help butterflies to distinguish each other, define their territories, and attract and keep mates. UV signals are just one piece of this much larger puzzle.

**Figure 3:**
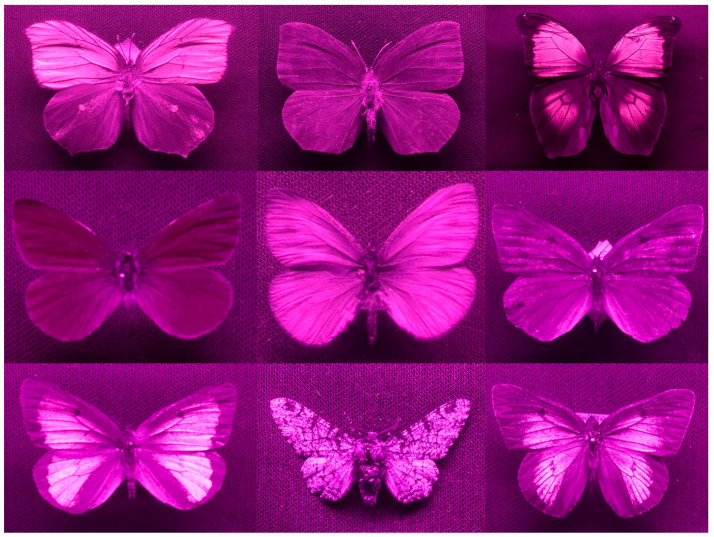
A relatively large proportion of studies fails to consider the widespread UV phenomena and many researchers neglect the UV spectrum as part of the integrated visual system.

This, however, is only a small part of the UV umwelt briefly outlined above, an umwelt that encompasses a variety of other ecological and evolutionary factors. UV patches may play a significant role in intraspecific and/or interspecific communication of Lepidoptera and we believe they are also a functional and effective taxonomical tool. The ubiquity of UV photoreceptors in the visual systems of many animals shows that UV colour is as important as other visible colours. Studies that quantify colour, or even qualitatively describe or compare colour patterns, must take UV colour into account. Descriptions of colour should not merely reflect our view of them. They should be set up with full consideration for the purpose of colour. A relatively large proportion of studies fails to account for UV phenomena and many researchers neglect the UV spectrum as part of the integrated visual system of many animals (Figure 3). On the other hand, there is no reason why one should assign a higher value to UV patterns than to the wing patterns visible to the human eye. As well, there is no good reason why we should assume a special function for UV wing patterns as a signal in visual communication.

There is still much work to be done in research on the manifold aspects of communication in the ultraviolet: ambient UV, UV patterns, UV vision, and finally, UV signal processing, and all the other yet undiscovered features that drives the animal UV umwelt. If we ignore these dimensions and study colour anthropocentrically, we introduce fatal flaws into our experiments, as we do when we fail to represent all parts of the hidden UV world.

## Data Availability

This review did not report any data.

## References

[1] Silberglied R.E., Taylor J., Orley R. (1978). Ultraviolet reflection and its behavioral role in the courtship of the sulphur butterflies *Colias eurytheme* and *C. philodice* (Lepidoptera, Pieridae). Behav. Ecol. Sociobiol..

[2] Lutz F.E. (1933). “Invisible” colors of flowers and butterflies. Nat. Hist..

[3] Crane J. (1954). Spectral reflectance characteristics of butterflies (Lepidoptera) from Trinidad, BWI. Zoologica.

[4] Meyer-Rochow V.B., Järvilehto M. (1997). Ultraviolet Colours in *Pieris napi* from Northern and Southern Finland: Arctic Females Are the Brightest!. Naturwissenschaften.

[5] Meyer-Rochow V.B. (1983). Flugelfarben, wie sie die Falter sehen—A study of UV-and other colour patterns in Lepidoptera. Annot. Zool. Jpn..

[6] Lyytinen A., Lindström L., Mappes J. (2004). Ultraviolet reflection and predation risk in diurnal and nocturnal Lepidoptera. Behav. Ecol..

[7] Bowden W.B., Watt S.R. (1966). Chemical phenotypes of pteridine colour forms in *Pieris* butterflies. Nature.

[8] Silberglied R.E., Taylor O.R. (1973). Ultraviolet differences between the sulphur butterflies, *Colias eurytheme* and *C. philodice*, and a possible isolating mechanism. Nature.

[9] Nekrutenko Y.P. (1965). ’Gynandromorphic Effect‘ and the Optical Nature of Hidden Wing-pattern in *Gonepteryx rhamni* L. (Lepidoptera, Pieridae). Nature.

[10] Allyn A.C., Downey J.C. (1977). Observations on male U-V reflectance and scale ultrastructure in *Phoebis* (Pieridae). Bull. Allyn Mus..

[11] Kemp D.J. (2006). Heightened phenotypic variation and age-based fading of ultraviolet butterfly wing coloration. Evol. Ecol. Res..

[12] Kemp D.J., Macedonia J.M. (2006). Structural ultraviolet ornamentation in the butterfly *Hypolimnas bolina* L. (Nymphalidae): Visual, morphological and ecological properties. Aust. J. Zool..

[13] Dushkina N., Erten S., Lakhtakia A. (2017). Coloration and Structure of the Wings of *Chorinea sylphina* Bates. J. Lepid. Soc..

[14] Imafuku M., Hirose Y., Takeuchi T. (2002). Wing colors of *Chrysozephyrus butterflies* (Lepidoptera; Lycaenidae): Ultraviolet reflection by males. Zool. Sci..

[15] Imafuku M. (2008). Variation in UV light reflected from the wings of *Favonius* and *Quercusia* butterflies. Entomol. Sci..

[16] Huxley J. (1975). The basis of structural colour variation in two species of *Papilio*. J. Entomol. Ser. A Gen. Entomol..

[17] Eguchi E., Meyer-Rochow V.B. (1983). Ultraviolet photography of forty-three species of Lepidoptera representing ten families. Annot. Zool. Jpn..

[18] Vukusic P., Sambles J.R. (2003). Photonic structures in biology. Nature.

[19] Wilts B.D., Pirih P., Stavenga D.G. (2011). Spectral reflectance properties of iridescent pierid butterfly wings. J. Comp. Physiol. A.

[20] Ghiradella H. (2010). Insect cuticular surface modifications: Scales and other structural formations. Advances in Insect Physiology.

[21] Ghiradella H., Aneshansley D., Eisner T., Silberglied R.E., Hinton H.E. (1972). Ultraviolet reflection of a male butterfly: Interference color caused by thin-layer elaboration of wing scales. Science.

[22] Ren A., Day C.R., Hanly J.J., Counterman B.A., Morehouse N.I., Martin A. (2020). Convergent evolution of broadband reflectors underlies metallic coloration in butterflies. Front. Ecol. Evol..

[23] Ghiradella H. (1989). Structure and development of iridescent butterfly scales: Lattices and laminae. J. Morphol..

[24] Stavenga D.G., Giraldo M.A., Leertouwer H.L. (2010). Butterfly wing colors: Glass scales of *Graphium sarpedon* cause polarized iridescence and enhance blue/green pigment coloration of the wing membrane. J. Exp. Biol..

[25] Kemp D.J., Rutowski R.L. (2011). The role of coloration in mate choice and sexual interactions in butterflies. Advances in the Study of Behavior.

[26] Wilts B.D., IJbema N., Stavenga D.G. (2014). Pigmentary and photonic coloration mechanisms reveal taxonomic relationships of the Cattlehearts (Lepidoptera: Papilionidae: Parides). BMC Evol. Biol..

[27] Kemp D.J., Rutowski R.L. (2007). Condition dependence, quantitative genetics, and the potential signal content of iridescent ultraviolet butterfly coloration. Evolution.

[28] Stavenga D.G., Stowe S., Siebke K., Zeil J., Arikawa K. (2004). Butterfly wing colours: Scale beads make white pierid wings brighter. Proc. R. Soc. Lond. Ser. B Biol. Sci..

[29] Kumazawa K., Tanaka S., Negita K., Tabata H. (1994). Fluorescence from wing of *Morpho sulkowskyi* butterfly. Jpn. J. Appl. Phys..

[30] Wijnen B., Leertouwer H., Stavenga D. (2007). Colors and pterin pigmentation of pierid butterfly wings. J. Insect Physiol..

[31] Grether G.F., Hudon J., Endler J.A. (2001). Carotenoid scarcity, synthetic pteridine pigments and the evolution of sexual coloration in guppies (*Poecilia reticulata*). Proc. Biol. Sci..

[32] Rutowski R.L., Macedonia J.M., Morehouse N., Taylor-Taft L. (2005). Pterin pigments amplify iridescent ultraviolet signal in males of the orange sulphur butterfly, *Colias eurytheme*. Proc. R. Soc. B Biol. Sci..

[33] Morehouse N.I., Vukusic P., Rutowski R. (2007). Pterin pigment granules are responsible for both broadband light scattering and wavelength selective absorption in the wing scales of pierid butterflies. Proc. Biol. Sci..

[34] Knuttel H., Fiedler K. (2001). Host-plant-derived variation in ultraviolet wing patterns influences mate selection by male butterflies. J. Exp. Biol..

[35] Griffith S.C., Parker T.H., Olson V.A. (2006). Melanin-versus carotenoid-based sexual signals: Is the difference really so black and red?. Anim. Behav..

[36] Glover B.J., Whitney H.M. (2010). Structural colour and iridescence in plants: The poorly studied relations of pigment colour. Ann. Bot..

[37] Yoshioka S., Kinoshita S. (2004). Wavelength-selective and anisotropic light-diffusing scale on the wing of the *Morpho* butterfly. Proc. Biol. Sci..

[38] Rutowski R.L., Macedonia J.M., Kemp D.J., Taylor-Taft L. (2007). Diversity in structural ultraviolet coloration among female sulphur butterflies (Coliadinae, Pieridae). Arthropod Struct. Dev..

[39] Rutowski R.L., Macedonia J.M., Merry J.W., Morehouse N.I., Yturralde K., Taylor-Taft L., Gaalema D., Kemp D.J., Papke R.S. (2007). Iridescent ultraviolet signal in the orange sulphur butterfly (*Colias eurytheme*): Spatial, temporal and spectral properties. Biol. J. Linn. Soc..

[40] Endler J.A. (1992). Signals, signal conditions, and the direction of evolution. Am. Nat..

[41] Pirih P., Wilts B.D., Stavenga D.G. (2011). Spatial reflection patterns of iridescent wings of male pierid butterflies: Curved scales reflect at a wider angle than flat scales. J. Comp. Physiol. A.

[42] Vukusic P., Sambles J., Lawrence C., Wootton R. (2001). Now you see it-now you don‘t. Nature.

[43] Simpson R.K., McGraw K.J. (2018). It’s not just what you have, but how you use it: Solar-positional and behavioural effects on hummingbird colour appearance during courtship. Ecol. Lett..

[44] Smith J.M., Harper D. (2003). Animal Signals.

[45] Zahavi A. (1975). Mate selection—A selection for a handicap. J. Theor. Biol..

[46] Boggs C.L., Gilbert L.E. (1979). Male contribution to egg production in butterflies: Evidence for transfer of nutrients at mating. Science.

[47] Yang M., Pyornila A., Meyer-Rochow V.B. (2004). UV-reflectivity of parafocal eyespot elements on butterfly wings in normal and abnormal specimens. Entomol. Fenn..

[48] Sekimura T., Jenkins O.P. (2014). In Pattern Formation and Diversity in Butterfly Wings: Experiments and Models. Advances in Zoology Research.

[49] Stavenga D.G., Leertouwer H.L., Wilts B.D. (2014). Coloration principles of nymphaline butterflies—Thin films, melanin, ommochromes and wing scale stacking. J. Exp. Biol..

[50] Nijhout H.F. (1991). The Development and Evolution of Butterfly Wing Patterns.

[51] Krishna A., Nie X., Warren A.D., Llorente-Bousquets J.E., Briscoe A.D., Lee J. (2020). Infrared optical and thermal properties of microstructures in butterfly wings. Proc. Natl. Acad. Sci. USA.

[52] Caro T., Mallarino R. (2020). Coloration in Mammals. Trends Ecol. Evol..

[53] Gloger C.W.L. (1833). Das Abändern der Vögel durch Einfluss des Klima’s, etc.

[54] Ellers J., Boggs C.L. (2004). Evolutionary genetics of dorsal wing colour in *Colias* butterflies. J. Evol. Biol..

[55] Bishop T.R., Robertson M.P., Gibb H., Van Rensburg B.J., Braschler B., Chown S.L., Foord S.H., Munyai T.C., Okey I., Tshivhandekano P.G. (2016). Ant assemblages have darker and larger members in cold environments. Global Ecol. Biogeogr..

[56] Heidrich L., Friess N., Fiedler K., Brändle M., Hausmann A., Brandl R., Zeuss D. (2018). The dark side of Lepidoptera: Colour lightness of geometrid moths decreases with increasing latitude. Glob. Ecol. Biogeogr..

[57] Zhang L., Martin A., Perry M.W., van der Burg K.R., Matsuoka Y., Monteiro A., Reed R.D. (2017). Genetic Basis of Melanin Pigmentation in Butterfly Wings. Genetics.

[58] Tuomaala M., Kaitala A., Rutowski R. (2012). Females show greater changes in wing colour with latitude than males in the green-veined white butterfly, *Pieris napi* (Lepidoptera: Pieridae). Biol. J. Linn. Soc..

[59] Ramos M.E., Hulshof C.M. (2019). Using digitized museum collections to understand the effects of habitat on wing coloration in the Puerto Rican monarch. Biotropica.

[60] Shanks K., Senthilarasu S., Mallick T.K. (2015). White butterflies as solar photovoltaic concentrators. Sci. Rep..

[61] Endler J.A. (1991). Variation in the appearance of guppy color patterns to guppies and their predators under different visual conditions. Vis. Res..

[62] Petersen B., Toernblom O., Bodin N. (1951). Verhaltensstudien am Rapsweissling und Bergweissling (*Pieris napi* L. und *Pieris bryoniae* Ochs.). Behaviour.

[63] Stella D., Pecháček P., Meyer-Rochow V.B., Kleisner K. (2018). UV reflectance is associated with environmental conditions in Palaearctic *Pieris napi* (Lepidoptera: Pieridae). Insect Sci..

[64] Obara Y., Koshitaka H., Arikawa K. (2008). Better mate in the shade: Enhancement of male mating behaviour in the cabbage butterfly, *Pieris rapae crucivora*, in a UV-rich environment. J. Exp. Biol..

[65] Makino K., Satoh K., Koike M., Ueno N. (1952). Sex in *Pieris rapae* L. and the pteridin content of their wings. Nature.

[66] Wiernasz D.C. (1989). Female choice and sexual selection of male wing melanin pattern in *Pieris occidentalis* (Lepidoptera). Evolution.

[67] Silberglied R.E. (1979). Communication in the ultraviolet. Annu. Rev. Ecol. Syst..

[68] Lande R., Bradbury J.W., Andersson M.B. (1987). In Genetic Correlations Between the Sexes in the Evolution of Sexual Dimorphism and Mating Preferences. Sexual Selection: Testing the Alternatives.

[69] Turner J.R.G. (1978). Why male butterflies are non-mimetic: Natural selection, sexual selection, group selection, modification and sieving. Biol. J. Linn. Soc..

[70] Stride G.O. (1956). On the courtship behaviour of *Hypolimnas misippus* L.; (Lepidoptera, Nymphalidae), with notes on the mimetic association with *Danaus chrysippus* L.; (Lepidoptera, Danaidae). Br. J. Anim. Behav..

[71] Stride G.O. (1957). Investigations into the courtship behaviour of the male of *Hypolimnas misippus* L. (Lepidoptera, Nymphalidae), with special reference to the role of visual stimuli. Br. J. Anim. Behav..

[72] Rutowski R.L., Gilchrist G.W., Terkanian B. (1987). Female butterflies mated with recently mated males show reduced reproductive output. Behav. Ecol. Sociobiol..

[73] Bonduriansky R. (2001). The evolution of male mate choice in insects: A synthesis of ideas and evidence. Biol. Rev..

[74] Rutowski R.L., Gilchrist G.W. (1986). Copulation in *Colias eurytheme* (Lepidoptera: Pieridae): Patterns and frequency. J. Zool..

[75] Iwasa Y., Pomiankowski A. (1995). Continual change in mate preferences. Nature.

[76] White T.E., Zeil J., Kemp D.J. (2015). Signal design and courtship presentation coincide for highly biased delivery of an iridescent butterfly mating signal. Evolution.

[77] Hamilton W.D., Zuk M. (1982). Heritable true fitness and bright birds: A role for parasites?. Science.

[78] Kemp D.J. (2007). Female mating biases for bright ultraviolet iridescence in the butterfly *Eurema hecabe* (Pieridae). Behav. Ecol..

[79] Fitzpatrick S. (1998). Colour schemes for birds: Structural coloration and signals of quality in feathers. Ann. Zool. Fenn..

[80] Brooks R., Couldridge V. (1999). Multiple sexual ornaments coevolve with multiple mating preferences. Am. Nat..

[81] Grether G.F., Kolluru G.R., Nersissian K. (2004). Individual colour patches as multicomponent signals. Biol. Rev..

[82] Johnstone R.A. (1995). Sexual selection, honest advertisement and the handicap principle: Reviewing the evidence. Biol. Rev..

[83] Johnstone R.A., Reynolds J.D., Deutsch J.C. (1996). Mutual mate choice and sex differences in choosiness. Evolution.

[84] Schluter D., Price T. (1993). Honesty, perception and population divergence in sexually selected traits. Proc. R. Soc. Lond. Ser. B Biol. Sci..

[85] Lindsay W.R., Andersson S., Bererhi B., Höglund J., Johnsen A., Kvarnemo C., Leder E.H., Lifjeld J.T., Ninnes C.E., Olsson M. (2019). Endless forms of sexual selection. PeerJ.

[86] Doucet S.M., Montgomerie R. (2003). Multiple sexual ornaments in satin bowerbirds: Ultraviolet plumage and bowers signal different aspects of male quality. Behav. Ecol..

[87] Obara Y. (1970). Studies on the mating behavior of the White Cabbage Butterfly, *Pieris rapae crucivora* Boisduval. Z. Vgl. Physiol..

[88] Papke R.S., Kemp D.J., Rutowski R.L. (2007). Multimodal signalling: Structural ultraviolet reflectance predicts male mating success better than pheromones in the butterfly *Colias eurytheme* L. (Pieridae). Anim. Behav..

[89] Rutowski R.L. (1985). Evidence for mate choice in a sulphur butterfly (*Colias eurytheme*). Z. Tierpsychol..

[90] Nakagawa T., Eguchi E. (1994). Differences in Flicker Fusion Frequencies of the Five Spectral Photoreceptor Types in the Swallowtail Butterfly′ s Compound Eye. Zool. Sci..

[91] Takizawa T., Koyama N. (1974). Reflection of ultraviolet light from the wing surface of the cabbage butterfly, *Pieris rapae crucivora* Boisduval (Lepidoptera: Pieridae). J. Ser. A Biol..

[92] Coutsis J.G. (1996). Ultra-violet reflection pattern in *Polyommatus andronicus* Coutsis & Chávalas, 1995 and *Polyommatus icarus* (Rottenburg, 1775) (Lepidoptera: Lycaenidae). Phegea.

[93] Huq M., Bhardwaj S., Monteiro A. (2019). Male Bicyclus anynana Butterflies Choose Females on the Basis of Their Ventral UV-Reflective Eyespot Centers. J. Insect Sci..

[94] Sweeney A., Jiggins C., Johnsen S. (2003). Insect communication: Polarized light as a butterfly mating signal. Nature.

[95] Obara Y., Ozawa G., Fukano Y. (2008). Geographic variation in ultraviolet reflectance of the wings of the female cabbage butterfly, *Pieris rapae*. Zool. Sci..

[96] Costanzo K., Monteiro A. (2007). The use of chemical and visual cues in female choice in the butterfly *Bicyclus anynana*. Proc. Biol. Sci..

[97] Giraldo M., Stavenga D. (2007). Sexual dichroism and pigment localization in the wing scales of *Pieris rapae* butterflies. Proc. R. Soc. B Biol. Sci..

[98] Obara Y., Majerus M.N. (2000). Initial mate recognition in the British cabbage butterfly, *Pieris rapae rapae*. Zool. Sci..

[99] Obara Y., Watanabe K., Satoh T. (2010). UV reflectance of inter-subspecific hybrid females obtained by crossing cabbage butterflies from Japan (*Pieris rapae crucivora*) with those from New Zealand (*P. rapae rapae*). Entomol. Sci..

[100] Kral K. (2016). Implications of insect responses to supernormal visual releasing stimuli in intersexual communication and ower-visiting behaviour: A review. Eur. J. Entomol..

[101] Penn D.J., Számadó S. (2020). The Handicap Principle: How an erroneous hypothesis became a scientific principle. Biol. Rev..

[102] Andersson M.B. (1994). Sexual Selection.

[103] Morehouse N.I. (2014). Condition-dependent ornaments, life histories, and the evolving architecture of resource-use. Integr. Comp. Biol..

[104] Vane-Wright R.I., Vane-Wright R.I., Ackery P.R. (1984). The role of pseudosexual selection in the evolution of butterfly colour pattern. The Biology of Butterflies.

[105] Brunton C., Majerus M.N. (1995). Ultraviolet colours in butterflies: Intra-or inter-specific communication?. Proc. R. Soc. Lond. Ser. B Biol. Sci..

[106] Crane J. (1955). Imaginal behavior of a Trinidad butterfly, *Heliconius erato hydara* Heiwitson, with special reference to the social use of color. Zoologica.

[107] Merrill R.M., Dasmahapatra K.K., Davey J.W., Dell’Aglio D.D., Hanly J.J., Huber B., Jiggins C.D., Joron M., Kozak K.M., Llaurens V. (2015). The diversification of *Heliconius* butterflies: What have we learned in 150 years?. J. Evol. Biol..

[108] Dalbosco Dell’Aglio D. (2016). Behavioural and Ecological Interactions between *Heliconius* Butterflies, Their Predators and Host Plants. Ph.D. Thesis.

[109] Bybee S.M., Yuan F., Ramstetter M.D., Llorente-Bousquets J., Reed R.D., Osorio D., Briscoe A.D. (2011). UV photoreceptors and UV-yellow wing pigments in *Heliconius* butterflies allow a color signal to serve both mimicry and intraspecific communication. Am. Nat..

[110] Robertson K.A., Monteiro A. (2005). Female *Bicyclus anynana* butterflies choose males on the basis of their dorsal UV-reflective eyespot pupils. Proc. Biol. Sci..

[111] Dell’Aglio D.D., Troscianko J., McMillan W.O., Stevens M., Jiggins C.D. (2018). The appearance of mimetic *Heliconius* butterflies to predators and conspecifics. Evolution.

[112] Rutowski R.L., Nahm A.C., Macedonia J.M. (2010). Iridescent hindwing patches in the Pipevine Swallowtail: Differences in dorsal and ventral surfaces relate to signal function and context. Funct. Ecol..

[113] Tabata H., Hasegawa T., Nakagoshi M., Takikawa S., Tsusue M. (1996). Occurrence of biopterin in the wings of *Morpho* butterflies. Experientia.

[114] DeVries P.J., Penz C.M., Hill R.I. (2010). Vertical distribution, flight behaviour and evolution of wing morphology in *Morpho* butterflies. J. Anim. Ecol..

[115] Brunton C. (1998). The evolution of ultraviolet patterns in European *Colias* butterflies (Lepidoptera, Pieridae): A phylogeny using mitochondrial DNA. Heredity.

[116] Stella D., Faltýnek Fric Z., Rindoš M., Kleisner K., Pecháček P. (2018). Distribution of Ultraviolet Ornaments in *Colias* Butterflies (Lepidoptera: Pieridae). Environ. Entomol..

[117] Nekrutenko Y.P. (1968). Phylogeny and geographical distribution of the genus *Gonepteryx* (Lepidoptera, Pieridae): An attempt of study in historical zoogeography. Kiev Nauk. Dumka.

[118] Brunton C.F., Hurst G.D.D. (1998). Mitochondrial DNA phylogeny of Brimstone butterflies (genus *Gonepteryx*) from the Canary Islands and Madeira. Biol. J. Linn. Soc..

[119] Bozano G.C., Coutsis J.G., Herman P., Allegrucci G., Cesaroni D., Sbordoni V. (2016). Guide to the Butterflies of the Palearctic Region: Pieridae 3: Coliadinae: Rhodocerini, Euremini, Coliadini (Gonepteryx and others) & Dismorpiinae (Leptidea).

[120] Hanzalová D. (2018). Phylogeny of Brimstone Butterflies (genus Gonepteryx): The Evolution of Colour Pattern in UV Spectrum and Geographical Area. Faculty of Science. Master’s Thesis.

[121] Brown W.L., Wilson E.O. (1956). Character displacement. Syst. Zool..

[122] Graham S.M., Watt W.B., Gall L.F. (1980). Metabolic resource allocation vs. mating attractiveness: Adaptive pressures on the “alba” polymorphism of Colias butterflies. Proc. Natl. Acad. Sci. USA.

[123] Taylor O.R. (1973). Reproductive isolation in *Colias eurytheme* and *C. philodice* (Lepidoptera: Pieridae): Use of olfaction in mate selection. Ann. Entomol. Soc. Am..

[124] Meyer-Rochow V.B. (1991). Differences in ultraviolet wing patterns in the New Zealand lycaenid butterflies *Lycaena salustius*, *L. rauparaha*, and *L. feredayi* as a likely isolating mechanism. J. R. Soc. N. Z..

[125] Remington C.L. (1973). Ultraviolet reflectance in mimicry and sexual signals in the Lepidoptera. J. N. Y. Entomol. Soc..

[126] Nekrutenko Y.P. (1964). The hidden wing-pattern of some Palearctic species of *Gonepteryx* and its taxonomic value. J. Res. Lepid..

[127] Nekrutenko Y.P. (1965). Three cases of gynandromorphism in *Gonepteryx*: An observation with ultraviolet rays. J. Res. Lepid..

[128] Nekrutenko Y.P. (1970). New subspecies of *Gonepteryx rhamini* from Tian-Shan Mountains, USSR. Lepid. Soc. J..

[129] Nekrutenko Y.P. (1972). A new subspecies of *Gonepteryx amintha* (Pieridae) from Yunnan, Mainland China, with comparative notes. J. Res. Lepid..

[130] Pecháček P., Stella D., Keil P., Kleisner K. (2014). Environmental effects on the shape variation of male ultraviolet patterns in the Brimstone butterfly (*Gonepteryx rhamni*, Pieridae, Lepidoptera). Naturwissenschaften.

[131] Ferris C.D. (1972). Ultraviolet photography as an adjunct to taxonomy. Lepid. Soc. J..

[132] Schaider P. (1988). Unterschiede von *Lycaena hippothoe* und candens im UV-Licht (Lep., Lycaenidae). Atalanta.

[133] Ferris C.D. (1973). A revision of the *Colias alexandra* complex (Pieridae) aided by ultraviolet reflectance photography with designation of a new subspecies. J. Lepid. Soc..

[134] Ferris C.D. (1975). A note on films and ultraviolet photography. News Lepid. Soc..

[135] Wheat C.W., Watt W.B. (2008). A mitochondrial-DNA-based phylogeny for some evolutionary-genetic model species of *Colias* butterflies (Lepidoptera, Pieridae). Mol. Phylogenet. Evol..

[136] Gaunet A., Dincă V., Dapporto L., Montagud S., Vodă R., Schär S., Badiane A., Font E., Vila R. (2019). Two consecutive Wolbachia-mediated mitochondrial introgressions obscure taxonomy in Palearctic swallowtail butterflies (Lepidoptera, Papilionidae). Zool. Scr..

[137] Lyytinen A., Alatalo R.V., Lindström L., Mappes J. (1999). Are European white butterflies aposematic?. Evol. Ecol..

[138] Brues C.T. (1941). Photographic evidence on the visibility of color patterns in butterflies to the human and insect eye. Proc. Am. Acad. Arts Sci..

[139] Viitala J., Korplmäki E., Palokangas P., Koivula M. (1995). Attraction of kestrels to vole scent marks visible in ultraviolet light. Nature.

[140] Church S.C., Bennett A.T.D., Cuthill I.C., Hunt S., Hart N.S., Partridge J.C. (1998). Does lepidopteran larval crypsis extend into the ultraviolet?. Naturwissenschaften.

[141] Majerus M.E.N., Brunton C.F.A., Stalker J. (2000). A bird’s eye view of the peppered moth. J. Evol. Biol..

[142] Kettlewell H.B.D. (1965). Insect survival and selection for pattern. Science.

[143] Komárek S. (1998). Mimicry, Aposematism and Related Phenomena.

[144] Brower L.P., Ryerson W.N., Coppinger L.L., Glazier S.C. (1968). Ecological chemistry and the palatability spectrum. Science.

[145] Lyytinen A., Alatalo R.V., Lindström L., Mappes J. (2001). Can ultraviolet cues function as aposematic signals?. Behav. Ecol..

[146] Maddocks S.A., Church S.C., Cuthill I.C. (2001). The effects of the light environment on prey choice by zebra finches. J. Exp. Biol..

[147] Arias M., Mappes J., Desbois C., Gordon S., McClure M., Elias M., Nokelainen O., Gomez D. (2019). Transparency reduces predator detection in mimetic clearwing butterflies. Funct. Ecol..

[148] Murali G. (2018). Now you see me, now you don’t: Dynamic flash coloration as an antipredator strategy in motion. Anim. Behav..

[149] Kjernsmo K., Whitney H.M., Scott-Samuel N.E., Hall J.R., Knowles H., Talas L., Cuthill I.C. (2020). Iridescence as Camouflage. Curr. Biol..

[150] Prudic K.L., Stoehr A.M., Wasik B.R., Monteiro A. (2015). Eyespots deflect predator attack increasing fitness and promoting the evolution of phenotypic plasticity. Proc. R. Soc. B Biol. Sci..

[151] Olofsson M., Vallin A., Jakobsson S., Wiklund C. (2010). Marginal eyespots on butterfly wings deflect bird attacks under low light intensities with UV wavelengths. PLoS ONE.

[152] Dong C.M., McLean C.A., Moussalli A., Stuart-Fox D. (2019). Conserved visual sensitivities across divergent lizard lineages that differ in an ultraviolet sexual signal. Ecol. Evol..

[153] Hastad O., Victorsson J., Odeen A. (2005). Differences in color vision make passerines less conspicuous in the eyes of their predators. Proc. Natl. Acad. Sci. USA.

[154] Mullen P., Pohland G. (2008). Studies on UV reflection in feathers of some 1000 bird species: Are UV peaks in feathers correlated with violet-sensitive and ultraviolet-sensitive cones?. IBIS.

[155] Cummings M.E., Rosenthal G.G., Ryan M.J. (2003). A private ultraviolet channel in visual communication. Proc. Biol. Sci..

[156] Siebeck U.E., Parker A.N., Sprenger D., Mäthger L.M., Wallis G. (2010). A species of reef fish that uses ultraviolet patterns for covert face recognition. Curr. Biol..

[157] Le Roy C., Debat V., Llaurens V. (2019). Adaptive evolution of butterfly wing shape: From morphology to behaviour. Biol. Rev..

[158] Advani N.K., Parmesan C., Singer M.C. (2019). Takeoff temperatures in *Melitaea cinxia* butterflies from latitudinal and elevational range limits: A potential adaptation to solar irradiance. Ecol. Entomol..

[159] Chen Z., Xu L., Li L., Wu H., Xu Y. (2019). Effects of constant and fluctuating temperature on the development of the oriental fruit moth, *Grapholita molesta* (Lepidoptera: Tortricidae). Bull. Entomol. Res..

[160] Galarza J.A., Dhaygude K., Ghaedi B., Suisto K., Valkonen J., Mappes J. (2019). Evaluating responses to temperature during pre-metamorphosis and carry-over effects at post-metamorphosis in the wood tiger moth (*Arctia plantaginis*). Philos. Trans. R. Soc. B.

[161] Sekimura T., Nijhout H.F. (2017). Diversity and Evolution of Butterfly Wing Patterns.

[162] Brehm G., Zeuss D., Colwell R.K. (2019). Moth body size increases with elevation along a complete tropical elevational gradient for two hyperdiverse clades. Ecography.

[163] Montejo-Kovacevich G., Smith J.E., Meier J.I., Bacquet C.N., Whiltshire-Romero E., Nadeau N.J., Jiggins C.D. (2019). Altitude and life-history shape the evolution of Heliconius wings. Evolution.

[164] Hovanitz W. (1944). The ecological significance of the color phases of *Colias chrysotheme* in North America. Ecology.

[165] Dalrymple R.L., Kemp D.J., Flores-Moreno H., Laffan S.W., White T.E., Hemmings F.A., Tindall M.L., Moles A.T. (2015). Birds, butterflies and flowers in the tropics are not more colourful than those at higher latitudes. Glob. Ecol. Biogeogr..

[166] Beerli N., Bärtschi F., Ballesteros-Mejia L., Kitching I.J., Beck J. (2019). How has the environment shaped geographical patterns of insect body sizes? A test of hypotheses using sphingid moths. J. Biogeogr..

[167] Hazel W.N. (1990). Sex-limited variability mimicry in the swallowtail butterfly *Papilio polyxenes* Fabr. Heredity.

[168] Mazokhin-Porshnyakov G.A. (1954). Ultraviolet radiation of the sun as a factor in insect habitats. Zh. Obshchei. Biol..

[169] Koski M.H., Ashman T. (2015). Floral pigmentation patterns provide an example of Gloger’s rule in plants. Nat. Plants.

[170] Pecháček P., Stella D., Kleisner K. (2019). A morphometric analysis of environmental dependences between ultraviolet patches and wing venation patterns in *Gonepteryx* butterflies (Lepidoptera, Pieridae). Evol. Ecol..

[171] Fukano Y., Satoh T., Hirota T., Nishide Y., Obara Y. (2012). Geographic expansion of the cabbage butterfly (*Pieris rapae*) and the evolution of highly UV-reflecting females. Insect Sci..

[172] Dalrymple R.L., Flores-Moreno H., Kemp D.J., White T.E., Laffan S.W., Hemmings F.A., Hitchcock T.D., Moles A.T. (2018). Abiotic and biotic predictors of macroecological patterns in bird and butterfly coloration. Ecol. Monogr..

[173] Beckmann M., Václavík T., Manceur A.M., Šprtová L., von Wehrden H., Welk E., Cord A.F. (2014). gl UV: A global UV-B radiation data set for macroecological studies. Methods Ecol. Evol..

[174] Zitko M. (2019). Ecological Factors Influencing Variability of Ultraviolet Colouration of Flowers. Master’s Thesis.

[175] Macedonia J.M. (2001). Habitat light, colour variation, and ultraviolet reflectance in the Grand Cayman anole, *Anolis conspersus*. Biol. J. Linn. Soc..

[176] Prudic K.L., Jeon C., Cao H., Monteiro A. (2011). Developmental plasticity in sexual roles of butterfly species drives mutual sexual ornamentation. Science.

[177] Slansky F., Feeny P. (1977). Stabilization of the rate of nitrogen accumulation by larvae of the cabbage butterfly on wild and cultivated food plants. Ecol. Monogr..

[178] Morehouse N.I., Rutowski R.L. (2010). Developmental responses to variable diet composition in a butterfly: The role of nitrogen, carbohydrates and genotype. Oikos.

[179] Mouchet S.R., Vukusic P., Constant R. (2018). Structural colours in lepidopteran scales. Advances in Insect Physiology.

[180] Kemp D.J. (2008). Resource-mediated condition dependence in sexually dichromatic butterfly wing coloration. Evol. Int. J. Org. Evol..

[181] Knüttel H., Fiedler K. (2000). On the use of ultraviolet photography and ultraviolet wing patterns in butterfly morphology and taxonomy. J. Lepid. Soc..

[182] McGraw K.J., Hill G.E. (2006). Mechanics of carotenoid-based coloration. Bird Coloration.

[183] Van der Kooi Casper J., Stavenga D.G., Arikawa K., Belušič G., Kelber A. (2021). Evolution of insect color vision: From spectral sensitivity to visual ecology. Annu. Rev. Entomol..

[184] Stavenga D.G., Arikawa K. (2006). Evolution of color and vision of butterflies. Arthropod Struct. Dev..

[185] Arikawa K. (2017). The eyes and vision of butterflies. J. Physiol..

[186] Carlson S.D., Chi C. (1979). The functional morphology of the insect photoreceptor. Annu. Rev. Entomol..

[187] Qiu X., Vanhoutte K.A.J., Stavenga D.G., Arikawa K. (2002). Ommatidial heterogeneity in the compound eye of the male small white butterfly, *Pieris rapae crucivora*. Cell Tissue Res..

[188] Meyer-Rochow V.B. (2019). Eyes and Vision of the Bumblebee: A Brief Review on how Bumblebees Detect and Perceive Flowers. J. Apic..

[189] Kelber A., Somanathan H. (2019). Spatial Vision and Visually Guided Behavior in Apidae. Insects.

[190] Menzel R., Backhaus W., Stavenga D.G., Hardie R.C. (1989). Color vision honey bees: Phenomena and physiological mechanisms. Facets of Vision.

[191] Koshitaka H., Kinoshita M., Vorobyev M., Arikawa K. (2008). Tetrachromacy in a butterfly that has eight varieties of spectral receptors. Proc. R. Soc. B Biol. Sci..

[192] Briscoe A.D., Bernard G.D., Szeto A.S., Nagy L.M., White R.H. (2003). Not all butterfly eyes are created equal: Rhodopsin absorption spectra, molecular identification, and localization of ultraviolet-, blue-, and green-sensitive rhodopsin-encoding mRNAs in the retina of *Vanessa cardui*. J. Comp. Neurol..

[193] Stalleicken J., Labhart T., Mouritsen H. (2006). Physiological characterization of the compound eye in monarch butterflies with focus on the dorsal rim area. J. Comp. Physiol. A.

[194] Sauman I., Briscoe A.D., Zhu H., Shi D., Froy O., Stalleicken J., Yuan Q., Casselman A., Reppert S.M. (2005). Connecting the navigational clock to sun compass input in monarch butterfly brain. Neuron.

[195] Rutowski R.L., Boggs C.L., Watt W.B., Ehrlich P.R. (2003). Visual ecology of adult butterflies. Butterflies: Ecology and Evolution Taking Flight.

[196] Simoncelli E.P., Olshausen B.A. (2001). Natural image statistics and neural representation. Annu. Rev. Neurosci..

[197] Baden T., Euler T., Berens P. (2019). Understanding the retinal basis of vision across species. Nat. Rev. Neurosci..

[198] Papiorek S., Junker R.R., Alves-dos-Santos I., Melo G.A.R., Amaral-Neto L.P., Sazima M., Wolowski M., Freitas L., Lunau K. (2016). Bees, birds and yellow flowers: Pollinator-dependent convergent evolution of UV patterns. Plant Biol..

[199] Tocco C., Dacke M., Byrne M. (2019). Eye and wing structure closely reflects the visual ecology of dung beetles. J. Comp. Physiol. A.

[200] Catalán A., Macias-Munoz A., Briscoe A.D. (2018). Evolution of sex-biased gene expression and dosage compensation in the eye and brain of *Heliconius* butterflies. Mol. Biol. Evol..

[201] Rutowski R.L., Warrant E.J. (2002). Visual field structure in the *Empress Leilia*, *Asterocampa leilia* (Lepidoptera, Nymphalidae): Dimensions and regional variation in acuity. J. Comp. Physiol. A.

[202] Briscoe A.D. (2008). Reconstructing the ancestral butterfly eye: Focus on the opsins. J. Exp. Biol..

[203] Pirih P., Arikawa K., Stavenga D.G. (2010). An expanded set of photoreceptors in the Eastern Pale Clouded Yellow butterfly, *Colias erate*. J. Comp. Physiol. A.

[204] Cuthill I.C., Partridge J.C., Bennett A.T.D., Church S.C., Hart N.S., Hunt S. (2000). Ultraviolet vision in birds. Adv. Study Behav..

[205] Cronin T.W., Bok M.J. (2016). Photoreception and vision in the ultraviolet. J. Exp. Biol..

[206] Briscoe A.D., Bybee S.M., Bernard G.D., Yuan F., Sison-Mangus M.P., Reed R.D., Warren A.D., Llorente-Bousquets J., Chiao C.C. (2010). Positive selection of a duplicated UV-sensitive visual pigment coincides with wing pigment evolution in *Heliconius* butterflies. Proc. Natl. Acad. Sci. USA.

[207] Merry J.W., Morehouse N.I., Yturralde K., Rutowski R.L. (2006). The eyes of a patrolling butterfly: Visual field and eye structure in the Orange Sulphur, *Colias eurytheme* (Lepidoptera, Pieridae). J. Insect Physiol..

[208] Finkbeiner S.D., Briscoe A.D. (2021). True UV color vision in a female butterfly with two UV opsins. J. Exp. Biol..

[209] Meyer-Rochow V.B., Kashiwagi T., Eguchi E. (2002). Selective photoreceptor damage in four species of insects induced by experimental exposures to UV-irradiation. Micron.

[210] Friberg M., Vongvanich N., Borg-Karlson A., Kemp D.J., Merilaita S., Wiklund C. (2008). Female mate choice determines reproductive isolation between sympatric butterflies. Behav. Ecol. Sociobiol..

